# Unique Research for Developing a Full Factorial Design Evaluated Liquid Chromatography Technique for Estimating Budesonide and Formoterol Fumarate Dihydrate in the Presence of Specified and Degradation Impurities in Dry Powder Inhalation

**DOI:** 10.1002/bmc.6062

**Published:** 2025-01-10

**Authors:** Lova Gani Raju Bandaru, Naresh Konduru, Leela Prasad Kowtharapu, Rambabu Gundla, Phani Raja Kanuparthy, Naresh Kumar Katari

**Affiliations:** ^1^ Department of Chemistry, GITAM School of Science GITAM Deemed to Be University Hyderabad India

**Keywords:** budesonide (epimer), degradation impurities, formoterol fumarate Dihydrate, liquid chromatography, quality by design

## Abstract

A simple LC method has been developed and validated for estimating budesonide (epimer B + A) and formoterol fumarate dihydrate in dry powder inhalation. The development results of this study make it very significant. The degradation and process impurities in EP and ChP were identified in addition to budesonide and formoterol fumarate. As of yet, no one has reported all impurities using a single method. It is a unique research because it analyzes APSD (Aerodynamic Particle Size Distribution), DDU (Delivered Dose Uniformity), BU (Blend Uniformity), Assay, and cleaning test samples. It enhances the quality of medicine and separates all organic impurities and isomers through a suitable stationary phase (YMC‐Pack Pro C18, 150 × 4.6 mm × 3 μm). We optimized the chromatographic conditions: Injection volume was 20 μL, and flow rate was 1.0 mL/min. The wavelength was optimized at 220 nm. After experimental and validation results. An example is A, which contains sodium dihydrogen orthophosphate monohydrate, sodium 1‐decane sulfonate, adjusted pH 3.0, and acetonitrile at a ratio of 80:20 (v/v), and B, which contains pH 3.0 buffer and acetonitrile at a ratio of 20:80 (v/v) respectively. In addition to being optimized, the test method was validated according to ICH Q2(R2).

## 1 Introduction

**FIGURE 1:**
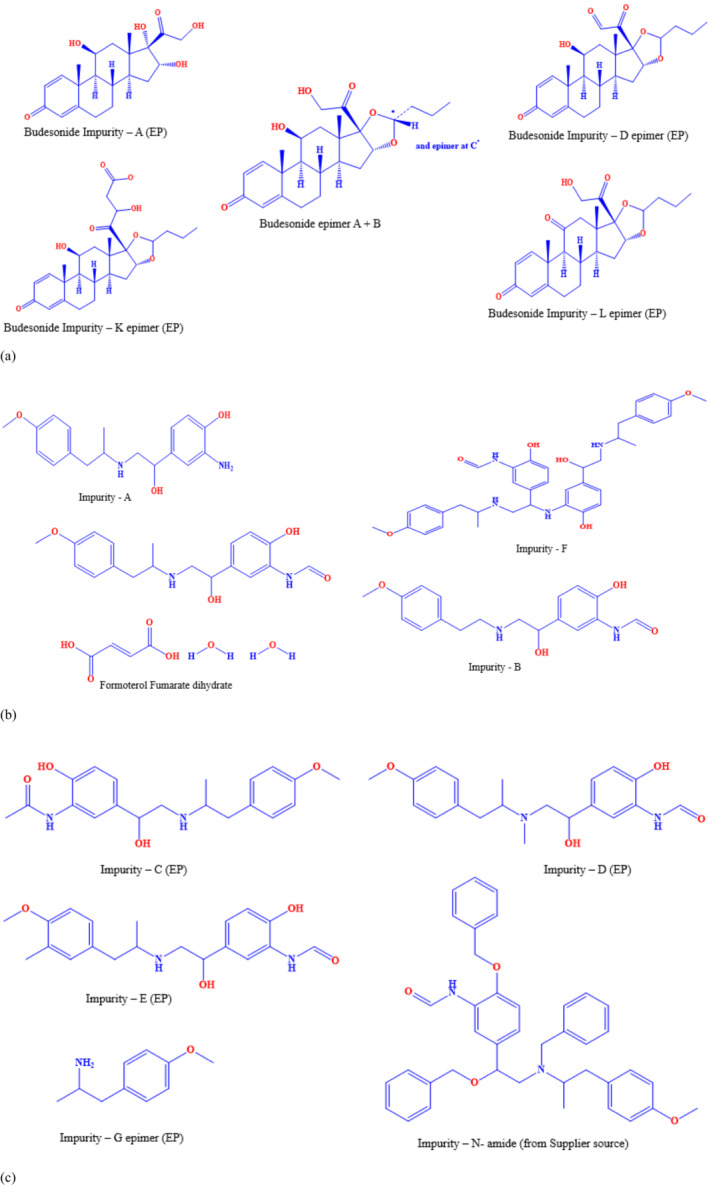
(a) Molecular structures of budesonide and their specified EP official monograph impurities. (b) Molecular structures of Formoterol Fumarate dihydrate and their specified EP official monograph impurities—A, F, B. (c) Molecular structures of Formoterol Fumarate dihydrate related specified EP official monograph impurities—C, D, E, G and suppler impurity.

An anti‐inflammatory substance, budesonide, is a steroid used to treat inflammation. The systemic absorption of budesonide is low compared to other glucocorticoids of the same generation. It treats asthma, rhinitis, and inflammatory bowel diseases (Agertoft, Pedersen, and Med 2000; Moore et al. 2013; Vestbo et al. 1999). A clinical trial is currently being conducted to evaluate the effectiveness of budesonide as a lung cancer prevention agent (Lam et al. 2004). The chemical formula is C_25_H_34_O_6,_ and the molecular weight is 430.53. Formoterol fumarate dihydrate is also known as formoterol (Tarsin, Assi, and Chrystyn 2004). The chemical formula is C_42_H_56_N_4_O_14_, and the molecular weight is 840.9. The budesonide (epimer B + A), formoterol fumarate dihydrate, and their specified impurities structures are shown in (Figure 1a–c). The budesonide and formoterol fumarate dihydrate aerosol are used for patients whose asthma cannot be adequately controlled by an inhaled corticosteroid (ICS) or for those whose asthma requires both ICS and long‐acting beta2‐adrenergic agonist (LABA**)** treatment (Slob et al. 2018). The targeted inhalation formulation does not have an official method in the United States Pharmacopeia (USP), British Pharmacopeia (BP), and Japanese Pharmacopeias (JP). An official monograph is available in Indian Pharmacopeia (IP) (Indian Pharmacopeia 2010). A literature survey revealed that few articles were reported for the individual content determination of budesonide (Hochhaus et al. 1998; Hryniewicka, Starczewska, and Golebiewska 2019; Demurtas et al. 2021; De et al. 2010; Varshosaz et al. 2012; Naikwade et al. 2009) and formoterol fumarate dihydrate (Akapo and Asif 2003; El‐Bagary et al. 2016; Trivedi, Chendake, and Patel 2012; Gondhale and Cheriyan 2022; Boltia et al. 2024; Salem et al. 2019). A few simultaneous determination articles for both APIs are published in various journals (Salem et al. 2017; Alkhateeb et al. 2021; Kale et al. 2014; Assi, Tarshin, and Chrystyn 2006; Jaybhaye and Singh 2021).

**FIGURE 2:**
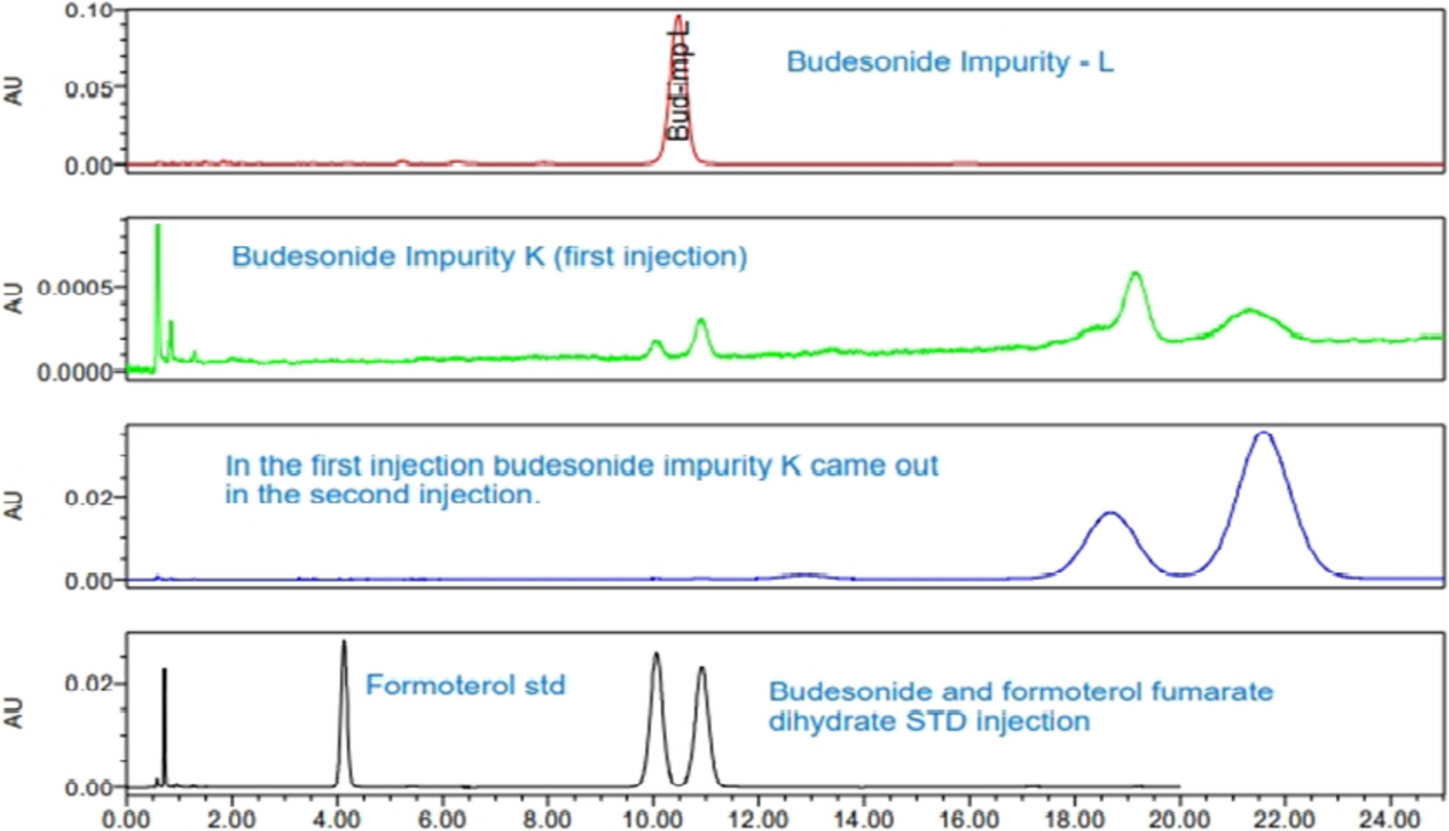
Overlay of budesonide Impurity L, Impurity K first injection, and second injection. In addition, budesonide and formoterol fumarate dihydrate are standard injections.

The suitability experiments were conducted using the reported methods and found that they are not suitable for the current study due to the coelution of budesonide major degradant impurity‐L at the retention time of budesonide Isomeric peak pair, and budesonide impurity‐K and formoterol fumarate dihydrate impurity‐F were not eluted within the analysis time (25 min) and eluted in subsequent blank injections. The coelution of budesonide impurity‐L and firmly retaining budesonide impurity‐K and formoterol fumarate dihydrate impurity‐F in the analytical column resulted in failure of method specificity (peak purity) and stability indicating nature. The specificity experiment chromatograms are shown in (Figure 2). The reported method's suitability experiments concluded that developing a specific stability‐indicating analytical method is mandatory to determine budesonide and formoterol fumarate dihydrate simultaneously. The major challenge of the current study is the separation of four budesonide impurities and eight formoterol fumarate dihydrate impurities from the active budesonide and formoterol fumarate dihydrate.

The product (Dry powder Inhalation) is sensitive to all degradation conditions; there is a chance of numerous degradant impurities throughout the shelf life. The impurities should not co‐elute with analytes; both analytes must be quantified simultaneously using a single method. The method should be able to determine budesonide and formoterol fumarate dihydrate from in‐process, finished, and stability samples. These challenges are targeted in the current research study to fulfill the set objectives (Table 1).

**TABLE 1:** Comparison of existing analytical methods and proposed test method.

Sample name/details	Stationary phase/pump mode	Observations/disadvantages	References
Formoterol Fumarate and Budesonide in Inhalation Suspensions	Column: Hypersil ODS (125 × 4.0) mm, 5 μm (thermos scientific)/isocratic mode (15 min)	The author did not discuss degradation impurities. The author did not prove a stability‐indicating method The author did not discuss specified impurities and their separation. The method cannot be helpful to the pharmaceutical industry.	Ravindra K Kotak et. Al DOI: 10.52711/0974‐360X.2021.00761.
Symbicort® metered dose inhaler	Column: BEH C18 (2.7 mm × 1.7 μm), 10 cm/gradient mode (25 min)	The author did not discuss formoterol fumarate‐specified and degradation impurities. The author did not discuss forced degradation studies. The author has developed a method of 25 min using the UPLC technique.	Fadi L. Alkhateeb et.al doi.org/10.1016/j.jpba.2020.113729.
Metered dose inhaler	Column: Hypersil BDS C‐18, 150 × 4.6 mm, 5 μm/gradient mode (30 min)	The author did not discuss formoterol fumarate and budesonide specified and degradation impurities. The author did not discuss peak purity details in the stressed samples. The method cannot be helpful to the pharmaceutical industry.	Nanasaheb et al. World Journal of Pharmaceutical Research, Volume 3, Issue 6, 1386–1399.
Metered dose inhaler	Column: Zorbax Eclipse XDB C18, (100 mm × 3 mm with 3.5 μm)/isocratic mode (15 min)	The author did not discuss formoterol fumarate and budesonide specified and degradation impurities. Table—IV shows alkaline degradation with 1 M NaOH on cold for 6 h; the percentage of degradation of FF is 57.4%, BUD epimer B is 45.5%, and BUD epimer A is 44.6%. Here, the author did not discuss degradation impurities. This test method did not show a stability‐indicating nature.	Y. A. Salem et al. doi: 10.1093/chromsci/bmx067.
Tablet dosage form	Column: Agilent (100 mm × 4.6 mm, 2.5 μm)/isocratic mode (15 min)	The author did not discuss formoterol fumarate and budesonide specified and degradation impurities. The author did not discuss the peak purity target compounds in the stressed degradation samples. In this method, all specified impurities cannot be identified.	Sunil Shivhari Jaybhaye et.al DOI: 10.15515/abr.0976‐4585.12.5.2331
Powder for Inhalation	IP monograph method Column: C18, (150 × 4.6) mm, 5 μm, (Waters, Spherisorb, ODS2)/isocratic mode (20 min)	The formoterol Imp – F1 and F2 did not come out within the runtime. The budesonide imp—K1 and K2 did not come out within the runtime. The budesonide imp—L came at budesonide epimer – B retention time. This IP monograph test method is not suitable to present regulatory compliance.	Indian Pharmacopeial IP 2010 Formoterol Fumarate and Budesonide Powder for Inhalation
Metered dose inhaler	TLC test method	The author did not explain degradation and known impurities information from this method. The author did not discuss forced degradation studies. This method is not suitable for the Quality Control department.	Mohammad Rizk et.al https://doi.org/10.1556/1006.2017.30.1.9

*Note:* Developed method: Using an LC instrument, the proposed test method was developed to estimate budesonide and formoterol fumarate dihydrate in the presence of various specified degradation impurities. For chromatography, the following conditions were used: (1) flow rate was set to 1.0 mL/min; (2) column was the YMC‐Pack Pro C18, 150 × 4.6 mm × 3 μm; (3) injection volume was set to 20 μL; and (4) a suitable gradient program was created for quality performance. The current test method separated eight formoterol fumarate known impurities, four budesonide‐related specified impurities, and known and unknown degradation impurities from the target compounds. Additionally, the mass balance was also achieved within the limits. Significant information: According to current regulatory guidelines, this method was developed. All validation parameters, including degradation studies, were verified using this method. Results have met specification limits. This method estimated budesonide and formoterol fumarate dihydrate in the Assay, Blend Uniformity, Content Uniformity, APSD, DDU, and cleaning testing samples. It can be used in Quality Control and Analytical Research Labs.

The statistical tool Quality by Design (QbD) concept is a modern technique that the regulatory body (ICH) suggested to use in the analytical method and formulation development (Konduru, Kowtharapu, and Gundla 2022; Susmitha, Rajitha, and Kumar 2023; Yenda et al. 2023; Konduru, Gundla, et al. 2022; Subramanian et al. 2020). The new guidelines are encouraging pharmaceutical industries to utilize the QbD tool. By using statistical, analytical, and risk‐management methodologies in the design, development, and manufacture of medicines, QbD aims to ensure quality. The current method utilizes this tool for evaluation by complete factorial design. Design Expert software version 13 was used in the current research.

We aimed to develop a specific, stability‐indicating, and robust analytical method using the Full Factorial Design to simultaneously determine budesonide and formoterol fumarate dihydrate from the dry powder inhalation formulation.

## 2 Materials and Methods

### 2.1 Chemicals and Reagents

Sodium dihydrogen orthophosphate monohydrate, sodium 1‐decane sulfonate, orthophosphoric acid (85%), water (Milli‐Q grade), hydrochloric acid (36.5–38.0%), sodium hydroxide (98%), hydrogen peroxide (30%), and acetonitrile (100%, Gradient grade) were supplied by Fisher Scientific (Mumbai, India). These chemicals were used to prepare the mobile phase, including diluent, standards, samples, and forced degradation samples. Vamsi Labs Ltd, Maharashtra, India, provided Budesonide (epimer), formoterol fumarate dihydrate, and their specific impurities. The finished product sample (Foracort 400 Rotacap) was procured from a local (Apollo) pharmacy in Hyderabad, Telangana, India.

### 2.2 Equipment and Software

This development work has been performed on RP‐HPLC (Waters Alliance e2695 HPLC system, Milford, MA 01757, USA) equipped with a quaternary pump and Photo‐diode array detector. Data acquisition and processing were made by using Empower‐3 software (Waters Corporations, Milford, MA, USA). The standards and samples were weighing on either XP4002S precision balance, XP205 Delta range analytical balance, AX205 Delta range analytical balance, or MX5 micro‐balance (Mettler Toledo, Columbus, OH, USA). The pH was measured using a SevenMulti pH meter (Mettler Toledo Columbus, OH, USA). Ultrasonic baths used were Branson 8510 (Emerson Electric, St. Louis, MO, USA). The photostability chamber was measured in a Suntest XLS + xenon test instrument (Atlas Material Testing Technology part of Ametek, Mount Prospect, IL, USA). A robust study used the Design‐Expert software version 13 (Stat‐ease Inc, Minneapolis, USA).

### 2.3 Chromatographic Conditions

Using the proposed test method, we have estimated budesonide (epimer) and formoterol fumarate dihydrate in powder. Further, an appropriate stationary phase (YMC Pack Pro C18, 150 mm × 4.6 mm, 3 μm) successfully separated all specified degradation impurities. It was found that impurities were separated depending on the ionic strength and pH of the mobile phase. Therefore, sodium dihydrogen orthophosphate monohydrate, sodium 1‐decane sulfonate, and pH 3.0 were selected. We prepared the mobile phase buffer, such as 1.38 g of sodium dihydrogen orthophosphate monohydrate and 1.22 g of 1‐decane sodium sulfonate, transferred into 1000 mL of milli‐q water to dissolve it and mixed well. Adjusted pH 3.0 with diluted orthophosphoric acid. We filtered the solution through a 0.22 μm of filter. Mobile phase A consisted of buffer and acetonitrile in 80:20 (v/v), and Mobile phase B contained pH 3.0 buffer and acetonitrile in the 20:80 (v/v) ratio, respectively. Measurements were made with an injection volume of 20 μL and a flow rate of 1.0 mL/min, a column temperature of 45°C, and ultraviolet (UV) detection was carried out at 220 nm. The mobile phase was passed with a proper gradient program: Time (minutes)/% solvent A: 0.0/80, 2.0/80, 15.0/65, 20.0/65, 22.0/30, 28.0/30, 30.0/80, and 35/80.

### 2.4 Diluent Preparation

The diluent was prepared with 65:35 (v/v) buffer and acetonitrile and then sonicated. As a result of method development and validation data, this diluent was chosen.

### 2.5 Formoterol Fumarate Dihydrate Standard Stock Solution Preparation

We accurately weighed and transferred 23.2 mg of formoterol fumarate dihydrate working standard into a 200 mL of volumetric flask, added about 120 mL of diluent, sonicated it to dissolve, and made up the volume with diluent (Standard stock solution concentration is 115 μg/mL).

### 2.6 Assay Standard Solution Preparation

We accurately weighed and transferred 20.1 mg of budesonide (epimer B + A) working standard into 100 mL of volumetric flask, added about 50 mL of diluent, sonicated to dissolve it, then spiked 5 mL of formoterol fumarate dihydrate standard stock solution into the flask, and made up to volume with diluent (The final assay standard solution concentration is as follows: 5.75 μg/mL of formoterol fumarate dihydrate and 200 μg/mL of budesonide epimer B + A).

### 2.7 Assay Sample Solution Preparation (Budesonide 194.15mcg/Formoterol Fumarate Dihydrate 5.85 mcg)

We weighed and transferred about 101.3 mg of sample powder into a 200 mL o volumetric flask and added about 120 mL of diluent, sonicated for 10 min to dissolve it. Then, it was made up to volume with diluent and mixed well. The sample solution was filtered through a 0.22 μm of Nylon filter paper. The final concentration is (5.85 μg/mL of formoterol fumarate dihydrate and 194.15 μg/mL of budesonide epimer B + A).

### 2.8 Placebo Solution Preparation (Without APIs)

We eeighed and transferred about 98.6 mg of placebo powder into a 200 mLof volumetric flask and added about 120 mL of diluent, sonicated for 10 min to dissolve it. Then, it was made up to volume with diluent and mixed well. The sample solution was filtered through a 0.22 μm Nylon filter paper.

### 2.9 Assay Spiked Sample Solution Preparation

We prepared a 5% level of all EP and ChP‐related specified impurities spiked sample solution properly. Each budesonide specified impurity of 10 mg was transferred into a 25 mL of volumetric flask, and 15 mL of diluent was added, appropriately sonicated, and made up to volume with diluent. Likewise, each formoterol fumarate dihydrate specified impurity of 6 mg was transferred into a 200 mL of volumetric flask, and 120 mL of diluent was added, appropriately sonicated, and made up to volume with diluent.

After being weighed accurately, 101.5 mg of sample powder was transferred into a 200 mL of volumetric flask, and 120 mL of diluent was added, appropriately sonicated, budesonide each impurity spiked 10 mL and formoterol fumarate dihydrate each impurity spiked 2 mL into sample volumetric flask, made up to volume with diluent, we have shaken it properly to mix the uniformity.

## 3 Results and Discussion

### 3.1 Method Development and Optimization

This research study aims to explain the critical and significant aspects of separating compounds with similar polarities from those with different properties and moieties. These parameters will be affected by column chemistry, mobile phase pH, and gradient programs. When a research study was conducted, we observed the budesonide, formoterol fumarate dihydrate, and their respective related impurity's solubility and physiochemical properties. As per experimental and literature data, both APIs have different behaviors. Before starting the analytical method development work, much literature was gathered from the various pharmacopeias (USP, EP, IP, and ChP), research articles, patents, and RLD product summary reports. The targeted inhalation formulation does not have an official monograph in the United States Pharmacopeia (USP), British Pharmacopeia (BP), Japanese Pharmacopeia (JP), and Chinese Pharmacopeia (ChP). An official monograph is available in Indian Pharmacopeia (IP). Based on the technical information, we concluded that the specific, precise, accurate, and robust method should be successfully developed and validated with proper evidence data.

**FIGURE 3:**
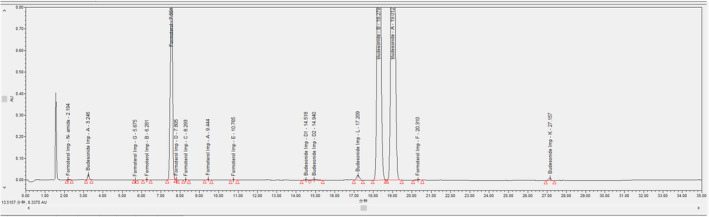
Spiked sample chromatogram (budesonide and formoterol fumarate dihydrate final test method).

Initially, we started the development trial using the Indian pharmacopeia test method. Due to the part of development work, the spiked sample solution was prepared and injected into the RP‐HPLC technique using the same test method. After experimental results were obtained, several issues were observed. Those are essential and need to be solved. Here, the major challenging works are (1) the separation between budesonide impurity‐L and budesonide API, (2) the separation between formoterol impurity‐D and formoterol fumarate dihydrate API, and (3) formoterol impurity‐F and budesonide impurity‐K should come within the runtime. A suitable stationary phase and gradient program solved these issues. In this research study, the leading role is a column (stationary phase) that has (YMC‐Pack Pro C18, 120 Å, 150 × 4.6 mm × 3 μm) a different column chemistry. The advantages are as follows: (1) It contains highly purified silica gel, followed by a high–coverage C18 bonding, and finishes with a unique end‐capping procedure utilizing Lewis's acid/Lewis base chemistry. These features provide unequaled separations with sharp and symmetrical peaks for all organic molecules, especially essential pharmaceuticals and chelating compounds. Moreover, it contains additional significant information, such as a carbon load of 16.0%, pH range of 2–8, pore size of 120 Å, and surface area of 330 m^2^/g. Most importantly, it helped separate similar polarity compounds. After separating well between degradation compounds, we conducted the forced degradation and validation studies using the final optimized test method. Based on the validation results, a stability‐indicating method was finally developed to estimate budesonide (epimers) and formoterol fumarate dihydrate in powder for inhalation drug products (Figure 3). The method optimization trails can be seen in (Table 2).

**TABLE 2:** Method optimization trails.

No. of experiments	Method details	Column	Results and Observations
Experiment 1	Mobile phase: Sodium phosphate buffer, pH 3.0: acetonitrile (65:35 v/v), flow rate: 2.0 mL/min, injection volume: 20 μL, UV: 220 nm, runtime: 20 min (isocratic mode).	(Waters, Spherisorb, ODS2) C18 column (150 mm × 4.6 mm, 5 μm), temp: 25°C	The formoterol impurity—F did not come out within the runtime. The budesonide impurity—K did not come out within the runtime. The budesonide impurity—L came at budesonide retention time. This method was taken from the IP monograph. This method was rejected.
Experiment 2	Mobile phase A: Sodium phosphate buffer with pH 3.0; mobile phase B: acetonitrile, flow rate: 2.0 mL/min, injection volume: 20 μL, UV: 220 nm, runtime: 35 min (gradient mode).	(Waters, Spherisorb, ODS2) C18 column (150 mm × 4.6 mm, 5 μm), temp: 25°C	The formoterol impurity—F1 and F2 came out within the runtime of 35 min (21.3 and 21.5 min). The budesonide impurity—K1 and K2 came out within the runtime of 35 min (20.5 and 20.6 min). The budesonide impurity—L came at budesonide epimer B retention time. This mobile phase strength was not enough. This method was rejected.
Experiment 3	Mobile phase A: Sodium phosphate buffer with pH 3.0 and acetonitrile (80:20) v/v; Mobile phase B: Buffer and acetonitrile (20:80) v/v respectively, flow rate: 1.0 mL/min, injection volume: 20 μL, UV: 220 nm, runtime: 35 min (gradient mode).	(Waters, Spherisorb, ODS2) C18 column (150 mm × 4.6 mm, 5 μm), temp: 25°C	The budesonide impurity—L came at budesonide epimer A retention time. This stationary phase needs to be changed. Because the column chemistry is not suitable for similar polarity compounds. This method was rejected.
Experiment 4	Mobile phase A: Sodium phosphate buffer with pH 3.0 and acetonitrile (80:20) v/v; Mobile phase B: Buffer and acetonitrile (20:80) v/v respectively, flow rate: 1.0 mL/min, injection volume: 20 μL, UV: 220 nm, runtime: 35 min (gradient mode).	YMC Pack Pro C18 (150 mm × 4.6 mm, 3.5 μm), temp: 45°C	The resolution between budesonide impurity—L and budesonide epimers A: **1.3** In this experiment, all specified impurities came out within the runtime and without interference. In this experiment, optimistic hope happened between Impurity L and budesonide API. So, the gradient program needs to be changed to separate them. This method was rejected.
Experiment 5	Mobile phase A: Sodium phosphate buffer with pH 3.0 and acetonitrile (80:20) v/v; Mobile phase B: Buffer and acetonitrile (20:80) v/v respectively, flow rate: 1.0 mL/min, injection volume: 20 μL, UV: 220 nm, runtime: 35 min (gradient mode).	YMC Pack Pro C18 (150 mm × 4.6 mm, 3.5 μm), temp: 45°C	The resolution between budesonide impurity—L and budesonide epimers A: **2.6** In this experiment, all specified impurities came out within the runtime and separated well. System suitability results were satisfactory. In the forced degradation sample, both APIs were peak purity passed. We conducted the validation studies on the proposed test method. This method was selected.

## 4 Method Validation

After optimization, the proposed test method has been validated according to ICH Q2 (R2) (ICH Q2 (R2). 2023) and USP < 1225 > guidelines. Moreover, we have performed degradation and stability studies to identify degradation routes and sensitivity. This method can be used for PK–BE clinical batch and stability studies analysis in the pharmaceutical industry's R&D and Quality Control labs based on the validation data.

### 4.1 System Suitability

We prepared the assay standard solution with a concentration of 6.0 μg/mL of formoterol fumarate dihydrate and 400 μg/mL of budesonide epimer B + A. After that, it was injected six times into the liquid chromatography technique. A reproducible result was obtained (Table 3).

**TABLE 3:** Method validation results.

Parameters	Details	Acceptance criteria	Results			
Specificity	*n* = 1, each individual injection for blank, standard, sample, and spiked sample with specified impurities.	No significant Interference of blank and placebo should be observed. No significant Interference from degradation products and impurities should be observed. Peak purity should pass for the analytes.	There are no peak interferences at the target compound retention time. Budesonide epimer A + B and Formoterol fumarate dihydrate peak purity passed in all degradation and spiked sample solutions.			
Precision (Day 1)	*n* = 6 (six determinations of assay sample solutions)	% RSD for replicate analysis should not be more than 2.0	Budesonide = 0.3% Formoterol fumarate dihydrate = 0.8%			
Precision (Day 2)	*n* = 6 (six determinations of assay sample solutions)	% RSD for replicate analysis between two analysts should not be more than 2.0	Budesonide = 0.7% Formoterol fumarate dihydrate = 0.9%			
Accuracy	*n* = 12 (three determinations, each level at 50, 100, 200, and 300% level of samples.	% analyte recovery should be 98.0 to 102% within a specified range. % of difference should not be more than 2.0	Budesonide epimer (A + B) results			
			50%	100%	200%	300%
			99.1	100.3	101.3	99.3
			99.5	100.1	100.1	99.8
			100.2	101.1	99.8	100.6
			0.56	0.53	0.79	0.66
			Formoterol fumarate dihydrate results			
			50%	100%	200%	300%
			99.6	100.2	99.8	100.8
			98.8	101.1	99.7	99.9
			99.2	99.6	101.2	99.6
			0.40	0.75	0.84	0.62
Linearity	*n* = 6 (six concentration levels in the range 20%–300%)	The correlation coefficient (*r* ^2^) should not be less than 0.99 Within the specified range.	Budesonide epimer (A + B) = 0.9997 Formoterol fumarate dihydrate = 0.9999			
LOQ and LOD concentrations	Budesonide epimer (A + B), Formoterol fumarate dihydrate	NA	LOQ concentration for budesonide = 9.0 μg/mL, LOD concentration for budesonide = 3.0 μg/mL, LOQ concentration for formoterol = 0.54 μg/mL, LOD concentration for formoterol = 0.18 μg/mL,			
			Budesonide epimer (A + B) results			
Solution stability	Established this study at (refrigerator conditions 2°C–8°C) and (room temperatures 15°C–25°C) in various time intervals such as 12, 24, 36, and 48.	The percentage of difference should not be more than 2.0	Storage condition	%of area difference (initial and 12 h)	% of area difference (initial and 24 h)	% of area difference (initial and 48 h)
			2°C–8°C	0.3	0.5	0.7
			15°C–25°C	0.4	0.9	1.3
			2°C–8°C	0.3	0.6	0.9
			15°C–25°C	0.5	0.7	1.4
			Formoterol fumarate dihydrate results			
			Storage condition	% of area difference (initial and 12 h)	% of area difference (initial and 24 h)	% of area difference (initial and 48 h)
			2°C–8°C	0.2	0.4	0.8
			15°C–25°C	0.3	0.5	0.9
			2°C–8°C	0.4	0.6	0.8
			15°C–25°C	0.6	0.8	1.3
System suitability results	The primary assay standard has been six times injected in the proposed test method	% RSD of six standard injections should not be more than 2.0. The USP plate count should not be less than 3000. The USP tailing factor should not be more than 2.0	Budesonide epimer (A + B) results			
			R.T (minutes)	USP tailing	USP Plate count	%RSD of six standards
			17.90, 18.59	1.2, 1.3	18,263, 19,892	0.5, 0.6
			Formoterol fumarate dihydrate results			
			7.12	1.12	13,563	0.4
Filter study	Unfiltered and filtered test samples have been injected into the proposed test method.	The difference between unfiltered and filtered samples should not be more than 2.0%	Budesonide epimer (A + B) results			
			Name of the filter	Assay results (%)	% of difference	
			Centrifuged	99.8	NA	
			0.22 μm Nylon 66	99.7	0.1	
			0.22 μm PVDF	99.6	0.2	
			0.22 μm PTFE	100.5	0.7	
			Formoterol fumarate dihydrate results			
			Name of the filter	Assay results (%)	% of difference	
			Cenrtifuged	100.3	NA	
			0.22 μm Nylon 66	99.9	0.4	
			0.22 μm PVDF	99.5	0.8	
			0.22 μm PTFE	101.2	0.9	
Forced degradation results for Budesonide	**Stress condition**	Purity angle < purity threshold	**Purity angle**	**Purity threshold**	**% Assay**	**Purity flag**
	Control sample		Budesonide ‐ B: 0.094 Budesonide ‐ A:0.087	Budesonide ‐ B: 0.247 Budesonide ‐ A:0.244	101.4	No
	The sample was prepared with 0.1 HCl, 2 h at benchtop		Budesonide ‐ B: 0.093 Budesonide ‐ A:0.086	Budesonide ‐ B: 0.248 Budesonide ‐ A: 0.245	97.1	No
	The sample was prepared with 0.1 NaOH, 2 h at benchtop		Budesonide ‐ B: 0.086 Budesonide ‐ A:0.080	Budesonide ‐ B: 0.246 Budesonide ‐ A: 0.243	92.7	No
	The sample was prepared with 5% H_2_O_2_, 2 h at benchtop		Budesonide ‐ B: 0.089 Budesonide ‐ A:0.082	Budesonide ‐ B: 0.246 Budesonide ‐ A: 0.243	97.1	No
	The sample was prepared with thermal 80°C for 24 h		Budesonide ‐ B: 0.095 Budesonide ‐ A:0.089	Budesonide ‐ B: 0.247 Budesonide ‐ A: 0.244	93.4	No
	The sample was prepared with Humidity 80RH for 24 h		Budesonide ‐ B: 0.096 Budesonide ‐ A:0.090	Budesonide ‐ B: 0.241 Budesonide ‐ A: 0.242	99.5	No
	The sample was prepared with photolytic at 7 days (ICH Q1B)		Budesonide ‐ B: 0.094 Budesonide ‐ A:0.091	Budesonide ‐ B: 0.341 Budesonide ‐ A: 0.361	99.8	No
Forced degradation results for formoterol fumarate dihydrate	**Stress condition**	Purity angle < purity threshold	**Purity angle**	**Purity threshold**	**% Assay**	**Purity flag**
	Control sample		0.073	0.241	99.4	No
	The sample was prepared with 0.1 HCl, 2 h at benchtop		0.054	0.231	91.9	No
	The sample was prepared with 0.1 NaOH, 2 h at benchtop		0.060	0.239	96.8	No
	The sample was prepared with 5% H_2_O_2_, 2 h at benchtop		0.062	0.235	101.6	No
	The sample was prepared with thermal 80°C for 24 h		0.048	0.226	100.8	No
	The sample was prepared with Humidity 80RH for 24 h		0.056	0.237	99.6	No
	The sample was prepared with photolytic at 7 days (ICH Q1B)		0.061	0.259	99.2	No

*Note:* % RSD, relative standard deviation; stress condition, degradation study condition; purity angle, target peaks purity angle; purity threshold, target peaks purity threshold; % assay, target peaks assay value in (%); purity flag, target peaks purity flag.

### 4.2 Precision

The proposed test method proved to be precise by using repeatability and ruggedness (Ettaboina, Katakam, and Dongala 2022; Ettaboina, Nakkala, and Chathalingath 2022; Ettaboina et al. 2024). It was possible to find reproducibility results for target compounds in this study. A repeatability test was conducted by injecting six freshly prepared drug product sample solutions containing 6.0 g/mL formoterol fumarate dihydrate and 400 g/mL budesonide epimer B + A on the same day and their six freshly prepared sample solutions containing the same concentration of each compound on different days and using different LC instruments by another analytical scientist. Each compound's mean relative standard deviation (RSD) value was at least 2.0%, confirming that this test method has acceptable precision and can be applied to different laboratory environments. The results are shown in (Table 3).

### 4.3 Specificity

#### 4.3.1 Interference Test

**FIGURE 4:**
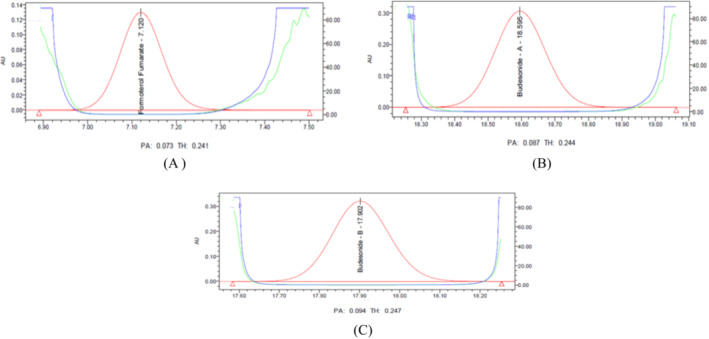
(A) Formoterol fumarate dihydrate purity index. (B) Budesonide epimer A Purity Index. (C) Budesonide epimer B purity index.

As an essential parameter in method confirmation, it can identify interference at the retention times of target compounds. Verification samples were injected into the proposed test method to determine the interference test (Konduru, Kethe, et al. 2022; Ettaboina and Nakkala 2021). These samples included blank, placebo, test as such, and test spiked samples with a 5% level of all specified impurities solutions. In addition, each specified impurity solution was injected into the LC instrument, and the retention time was observed. Reviewed all chromatography data; no interference was observed at retention times for budesonide epimer B + A and formoterol fumarate dihydrate. There is no discrepancy in the spectral purity (Figure 4A–C).

#### 4.3.2 Forced Degradation Studies

This study plays a significant role during the method development and validation stages because it can identify the stability‐indicating nature of the method as well as the behavior of the molecules under stress conditions (Ettaboina, Nakkala, and Chathalingath 2022). Before starting our experiments, we gathered information about stress conditions from API DMFs, research articles, patents, and drug product summaries. As reported in the literature, we conducted degradation studies using the following stress samples.

##### 4.3.2.1 Acid Degradation

**FIGURE 5:**
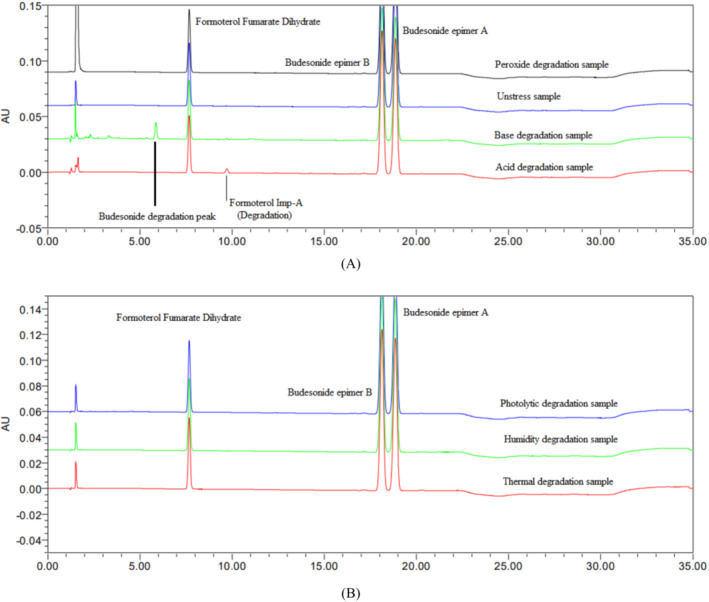
(A) Overlay of chemical degradation chromatograms. (B) Overlay of physical degradation chromatograms.

We accurately weighed and transferred 202.3 mg of sample powder into 200 mL of a volumetric flask, added 5 mL of 0.1 N HCl solution, and kept on the benchtop for 2 h. Abour 60 mL of diluent was added. The compounds were sonicated for 10 min to dissolve. Made up to volume with diluent, the solution was filtered through a 0.22 μm of Nylon66 filter, and the results are in (Table 3 and Figure 5A).

##### 4.3.2.2 Base Degradation

We accurately weighed and transferred 201.6 mg of sample powder into 200 mL of a volumetric flask, added 5 mL of 0.1 N NaOH solution, and kept on the benchtop for 2 h. About 60 mL of diluent was added. The compounds were sonicated for 10 min to dissolve. Made up to volume with diluent, the solution was filtered through a 0.22 μm of Nylon66 filter, and the results are in (Table 3 and Figure 5A).

##### 4.3.2.3 Oxidation Hydrolysis

We accurately weighed and transferred 202.1 mg of sample powder into 200 mL of a volumetric flask, added 5 mL of 5% H_2_O_2_ solution, and kept on the benchtop for 2 h. About 60 mL of diluent was added. The compounds were sonicated for 10 min to dissolve. Made up to volume with diluent, the solution was filtered through a 0.22 μm of Nylon66 filter, and the results are in (Table 3 and Figure 5A).

##### 4.3.2.4 Photolytic Condition

After exposing the sample to light according to the ICH Q1B (ICH Q1B. 1996) guideline's suggested conditions, the sample was prepared as follows: We accurately weighed and transferred 203.2 mg of sample powder into 200 mL of a volumetric flask, 60 mL of diluent was added, and the compounds were sonicated for 10 min to dissolve. Made up to volume with diluent, the solution was filtered through a 0.22 μm of Nylon66 filter; results are in (Table 3 and Figure 5B).

##### 4.3.2.5 Thermal Degradation

After exposing the sample to temperature (80°C for 24 h), the sample was prepared as follows. W accurately weighed and transferred 201.8 mg of sample powder into 200 mL of a volumetric flask, 60 mL of diluent was added, and the compounds were sonicated for 10 min to dissolve. Made up to volume with diluent, the solution was filtered through a 0.22 μm of Nylon66 filter; results are in (Table 3 and Figure 5B).

##### 4.3.2.6 Humidity Degradation

After exposing the sample to humidity (80% RH for 24 h), the sample was prepared as follows. W accurately weighed and transferred 202.4 mg of sample powder into 200 mL of a volumetric flask, 60 mL of diluent was added, and the compounds were sonicated for 10 min to dissolve. Made up to volume with diluent, the solution was filtered through a 0.22 μm of Nylon66 filter; results are in (Table 3 and Figure 5B).

In the final review of all degradation chromatograms, all degradation impurities were found within the runtime of the experiment. No interference was observed at target compound retention times. In all stressful conditions, there is no discrepancy in the spectral purity.

#### 4.3.3 Accuracy

**FIGURE 6:**
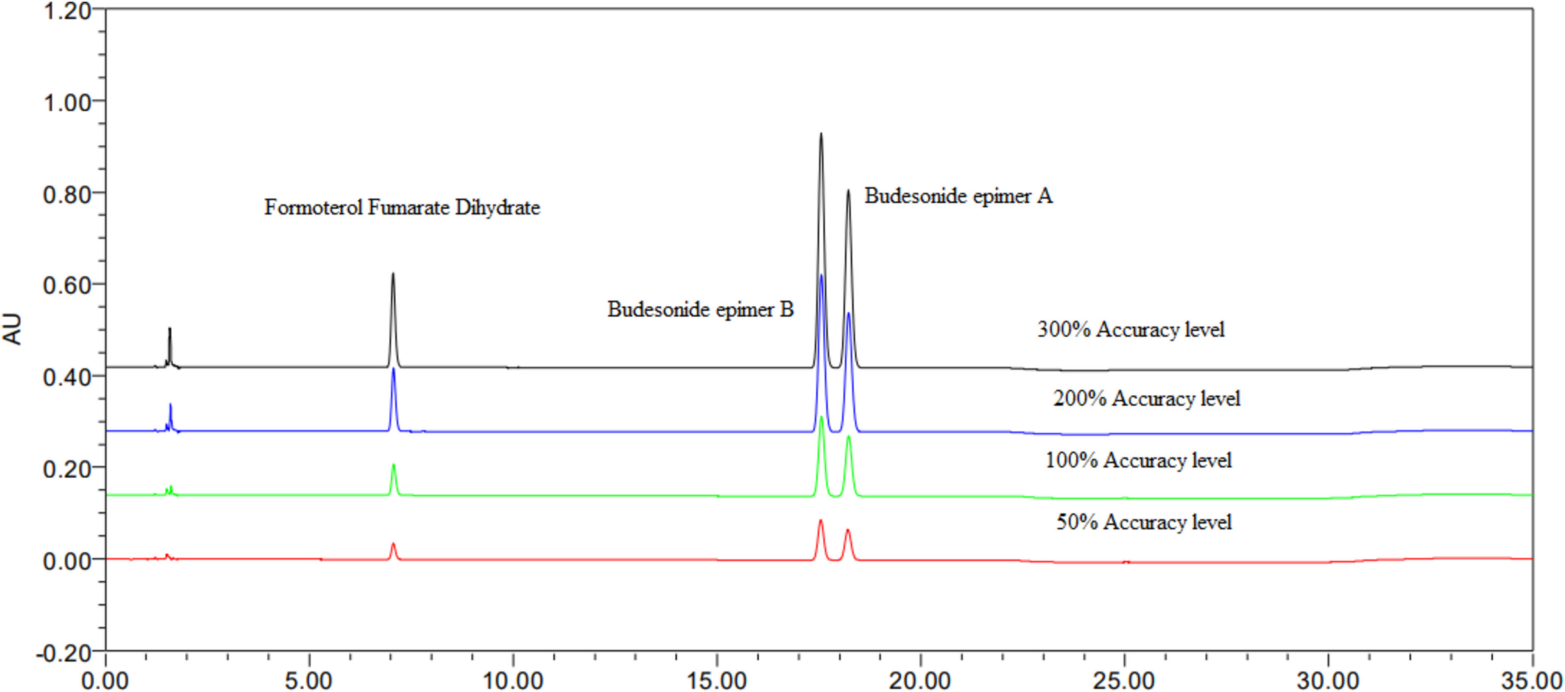
Overlay of various concentrations of accuracy samples.

The method's accuracy was proven by the standard spiking experiment to placebo. Here, both APIs and placebo spiked in the sample preparation. Spiked the API at various concentrations, such as 50%, 100%, 200%, and 300% with a constant placebo at 100%. Prepared recovery samples in triplicate and injected them into the LC system according to a proposed test method. Finally, the recovery results were obtained between 98.0% and 102%; the results are in (Table 3 and Figure 6).

#### 4.3.4 Linearity

The linearity study was performed from 20% to 300% with respective test concentrations. It is directly proportional to the analyte's attention and the analyte's response. These data from the regression line itself may be helpful in providing mathematical estimates of the degree of linearity; results are in (Table 3).

#### 4.3.5 LOQ and LOD Solutions Preparation

The LOQ and LOD solutions were established through the standard deviation of the response and the slope method procedure. The budesonide LOQ and LOD concentrations are 9 μg/mL/3.0 μg/mL; the formoterol fumarate dihydrate LOQ and LOD concentrations are 0.54 μg/mL/0.18 μg/mL. These concentrations were achieved from a linearity experiment, as shown in (Table 3).

#### 4.3.6 Solution Stability

This study is more advantageous for analytical scientists as it saves time and costs. Therefore, we have established standard and sample solution stability studies at 12, 24, and 48 h at room temperature (25 ± 2°C) and refrigerator storage conditions (2°C–8°C). Finally, we achieved results within the limits (< 2.0%), which can be seen in the results in (Table 3). The results show that budesonide and formoterol fumarate dihydrate are stable for up to 48 h at room temperature and in refrigerator conditions.

#### 4.3.7 Filter Compatibility Study

Filter compatibility experiment is mandatory for any analytical method; due to solid excipients in the current formulation, it is much needed to study in the current method. The filter study was conducted using three different filters such as 0.22 μm of Nylon66 filter, 0.22 μm of PVDF filter, and 0.22 μm PTFE filter. All three filters showed <2.0% assay difference against the centrifuged sample, and a 0.22 μm of Nylon66 filter was chosen for the regular analysis, which can be seen in the results in (Table 3).

#### 4.3.8 Robustness Study

The proposed method robustness was studied using the statical tool Design Expert software version 13. As mentioned in the optimization of the chromatography conditions, a buffer was selected from the IP needed to study the pH impact on the method. Based on the chromatography optimization process three critical method parameters (CMPs). The three CMPs were studied by utilizing the Design of Experiments (DoE's) in the light of the QbD concept. The selected three CMPs were Flow rate (0.1 mL/min), buffer pH (3.0 ± 0.2 units), and column temperature (45°C ± 5°C) (Vemuri et al. 2022; Muchakayala et al. 2023). The CMPs changed deliberately and constructed the design by considering three factors. Designed the Factorial DoEs with three factors, three center points with zero blocks, and three responses were recorded, which are resolution between FFD and FFD impurity D (R1), resolution between BDS impurity‐L and BDS (R2), and resolution between BDS and FFD impurity‐F (R3). A total of 19 runs were obtained from the design, and 19 DoE's ran on the HPLC, and the results were tabulated in (Table 4).

**TABLE 4:** DoE experiments and results.

Std	Run	Factor 1	Factor 2	Factor 3	Response 1	Response 2	Response 3
		A: Flow mL/min	B: Column temp°C	C: pH	R1	R2	R3
16	1	1.1	50	3.2	1.6	3.61	3.76
8	2	1.1	50	2.8	1.59	3.15	3.59
18	3	1	45	3	1.78	3.65	4.05
7	4	1.1	50	2.8	1.58	3.09	3.62
19	5	1	45	3	1.75	3.69	4.03
5	6	0.9	50	2.8	1.65	3.12	3.69
3	7	1.1	40	2.8	1.61	3.45	3.98
14	8	0.9	50	3.2	1.81	3.85	3.7
6	9	0.9	50	2.8	1.66	3.72	3.65
15	10	1.1	50	3.2	1.66	3.69	3.6
17	11	1	45	3	1.77	3.68	4.09
2	12	0.9	40	2.8	1.85	3.75	3.95
11	13	1.1	40	3.2	1.78	3.62	3.95
9	14	0.9	40	3.2	1.91	3.85	4.15
10	15	0.9	40	3.2	1.92	3.88	4.19
4	16	1.1	40	2.8	1.75	3.65	4.09
1	17	0.9	40	2.8	1.81	3.71	3.96
12	18	1.1	40	3.2	1.7	3.64	4.18
13	19	0.9	50	3.2	1.8	3.75	3.65

**FIGURE 7:**
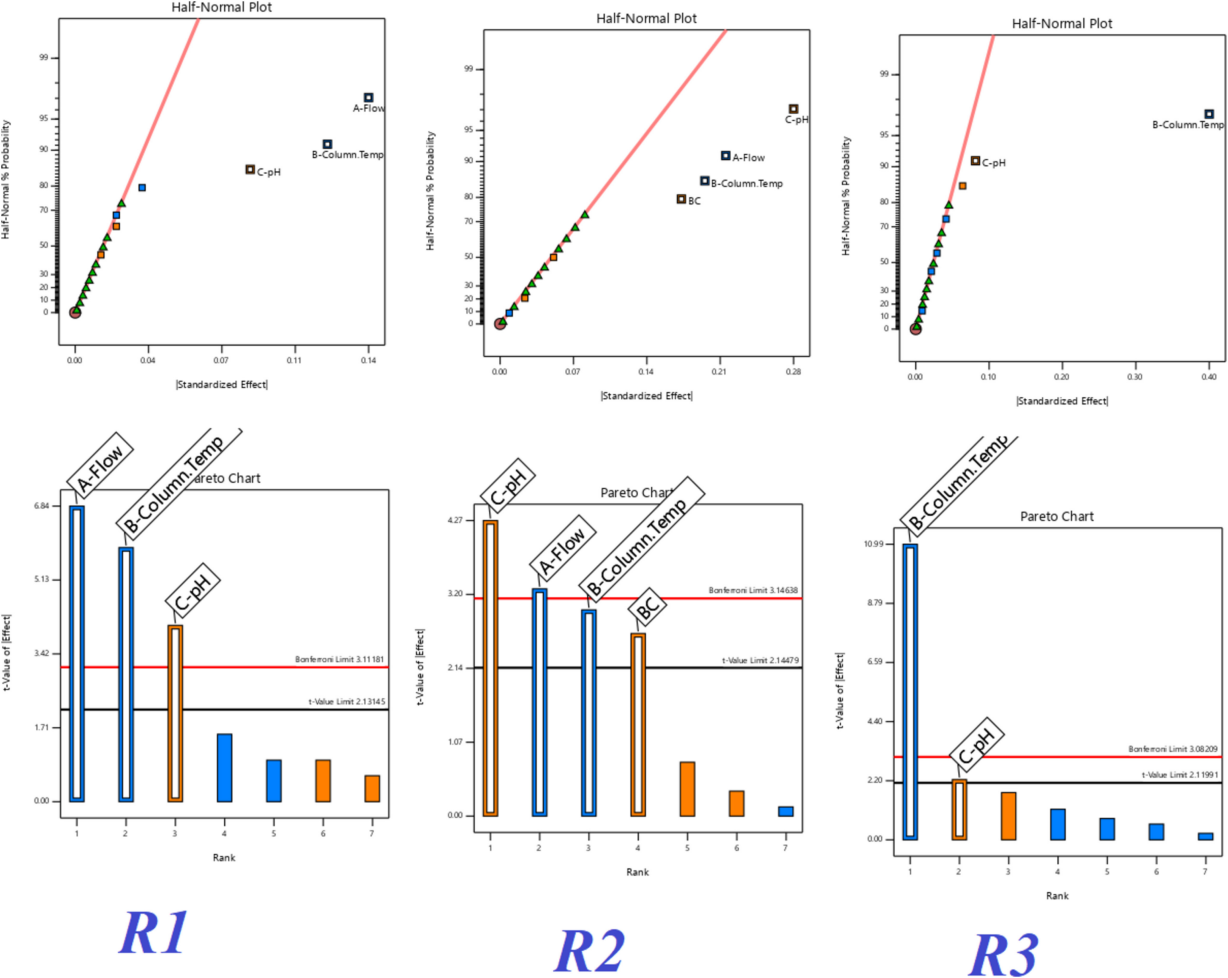
Quality by design half‐normal plots and Pareto charts.

**FIGURE 8:**
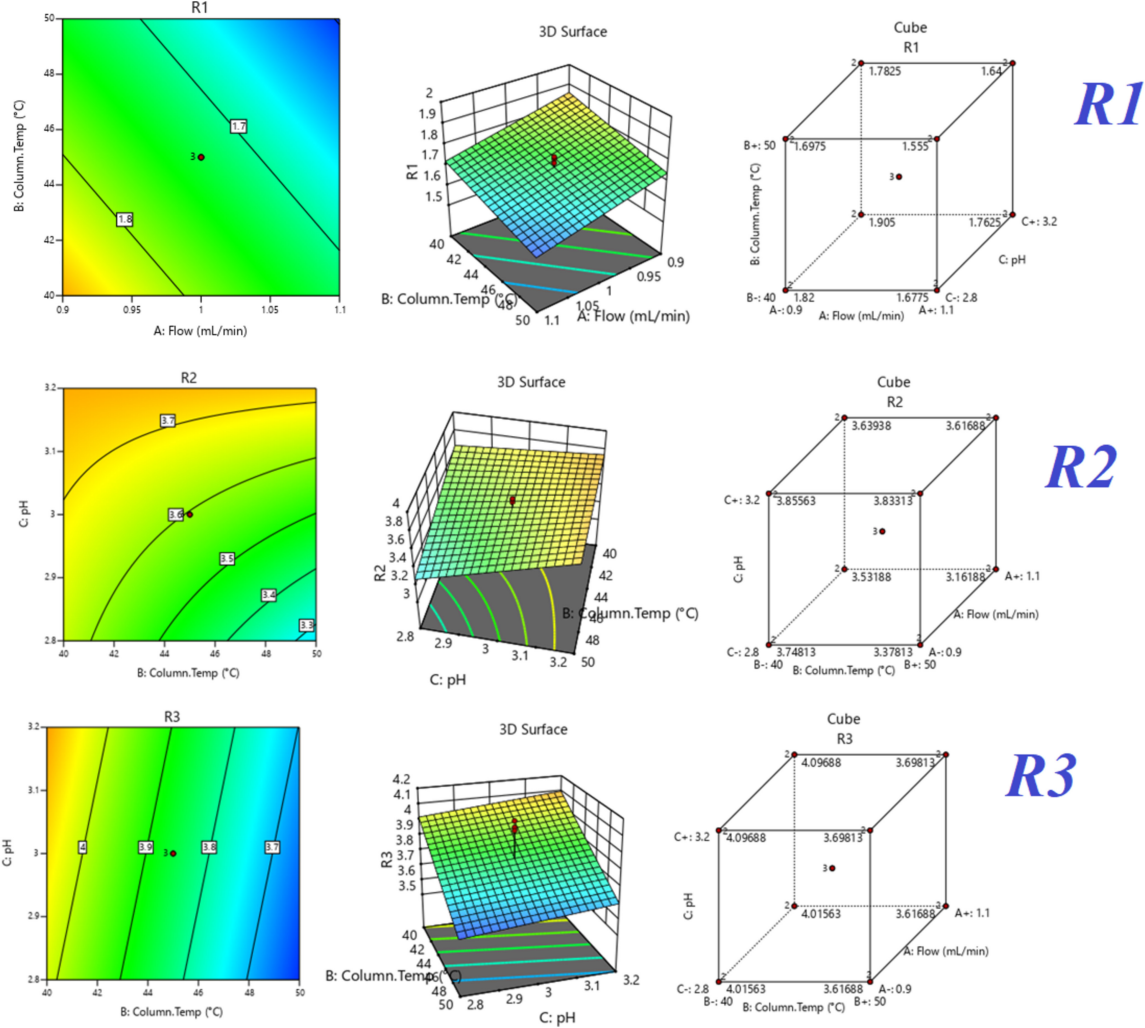
Quality by design 3D surface plots, 2D contour plots, and 3D cube plots.

**FIGURE 9:**
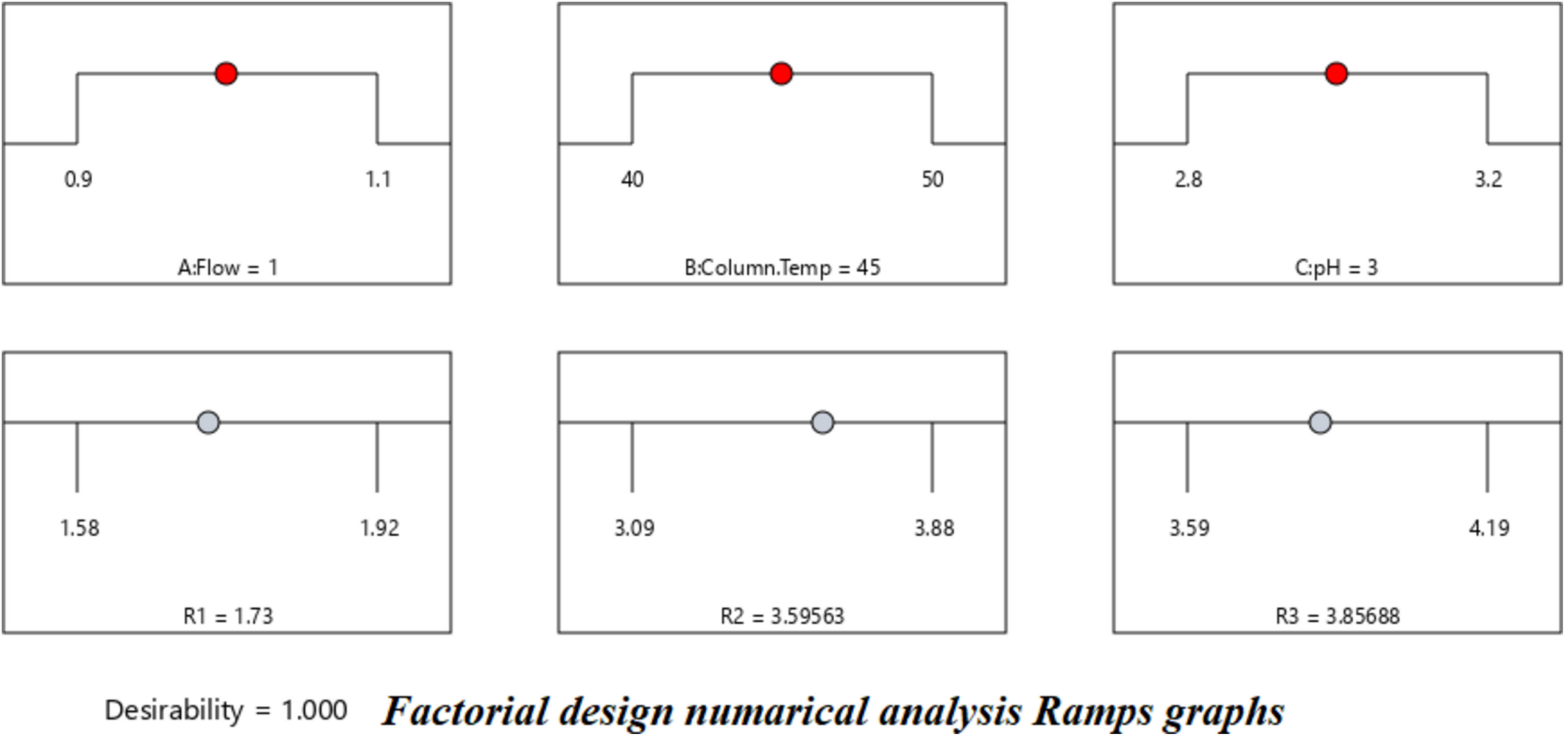
Factorial design numerical analysis ramps graphs.

**FIGURE 10:**
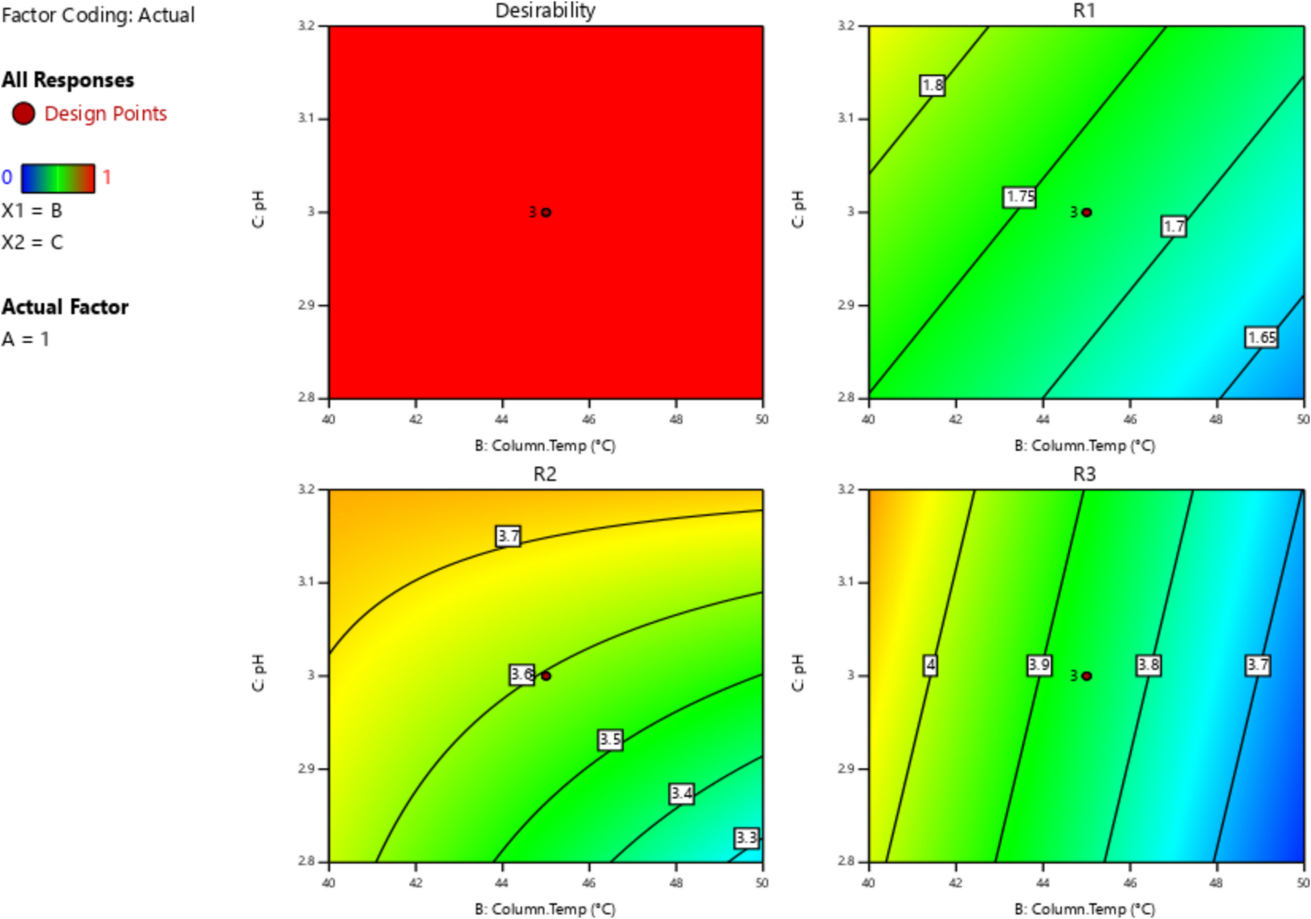
Factorial design desirability plots.

**FIGURE 11:**
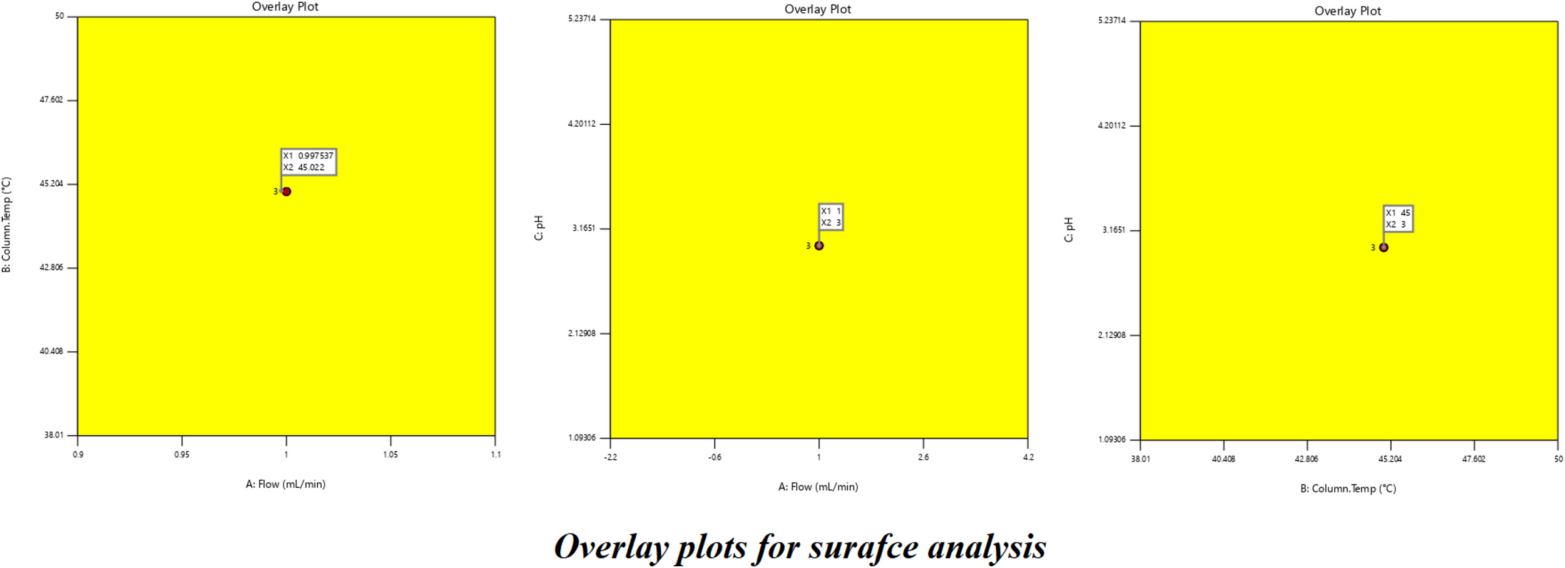
Factorial design overlay plots for surface analysis.

All three responses were analyzed by selecting the major effects from the actual. The major effect points are shown in Half normal plots and Pareto charts for three responses, R1, R2, and R3 (Figure 7), with no transform of linear regression. ANOVA table (Table 5) for selected factorial models R1, R2, and R3 shows the model is significant. The probability *p*‐value was shown below 0.05 for three responses. Adjusted *R* ^2^ and predicted *R* ^2^ values are shown below 0.2 difference for all three responses. Box–Cox diagnostics value shows 1 with no recommended transform. The 2D contour plots, 3D surface plots, and 3D cube surface plots are shown in (Figure 8). R1 and R2 showed a clear impact of all three factors, but the values were between 1.58 and 3.86, which was well within the limit > 1.5. R3 showed a clear impact of column temperature and pH, but the resolution was greater than 2.0. The changed deliberate variation did not show much impact on responses, and the method was found to be robust. The full factorial design numerical analysis results, desirability plots, and overlay plots are shown in (Figures 9, 10, 11).

**TABLE 5:** ANOVA table.

Response	Source	Sum of Squares	df	Mean Square	*F*‐value	*p*	
**R1**	**Model**	0.1702	3	0.0567	32.65	< 0.0001	Significant
	A‐flow	0.0812	1	0.0812	46.76	< 0.0001	
	B‐column temp	0.06	1	0.06	34.56	< 0.0001	
	C‐pH	0.0289	1	0.0289	16.64	0.0011	
	Curvature	0.0034	1	0.0034	1.96	0.1838	
	**Residual**	0.0243	14	0.0017			
	Lack of fit	0.008	4	0.002	1.24	0.3557	Not significant
	Pure error	0.0163	10	0.0016			
	**Cor total**	0.1979	18				
**R2**	**Model**	0.7783	4	0.1946	11.22	0.0004	Significant
	A‐Flow	0.1871	1	0.1871	10.79	0.0059	
	B‐column temp	0.1541	1	0.1541	8.89	0.0106	
	C‐pH	0.3164	1	0.3164	18.25	0.0009	
	bc	0.1208	1	0.1208	6.97	0.0204	
	Curvature	0.0153	1	0.0153	0.8799	0.3653	
	**Residual**	0.2254	13	0.0173			
	Lack of fit	0.0131	3	0.0044	0.2052	0.8905	Not significant
	Pure error	0.2123	10	0.0212			
	**Cor total**	1.02	18				
**R3**	**Model**	0.6624	2	0.3312	62.89	< 0.0001	Significant
	B‐column temp	0.636	1	0.636	120.76	< 0.0001	
	C‐pH	0.0264	1	0.0264	5.01	0.0407	
	Curvature	0.1008	1	0.1008	19.15	0.0005	
	**Residual**	0.079	15	0.0053			
	Lack of fit	0.0285	5	0.0057	1.13	0.4059	Not significant
	Pure error	0.0505	10	0.0051			
	**Cor total**	0.8423	18				

#### 4.3.9 Conclusion

This analytical test method was developed and validated for estimating budesonide epimer (A + B) and formoterol fumarate dihydrate in powder inhalation dosage form. All known and degraded impurities were separated from the target compounds. Therefore, this method was more helpful for estimating budesonide epimer and formoterol fumarate dihydrate in the Assay test, Blend Uniformity test, Aerodynamic Particle Size Distribution (APSD), Delivered Dose Uniformity (DDU), and cleaning test samples. We found a few degradation impurities in the stress samples; those had appeared during runtime. This method has been validated according to current regulatory guidelines (ICH Q2(R2) and USP 1225). Moreover, the robustness study was performed according to DOE trials, and the results were satisfactory. The validated test method detects 12 specified impurities. There has never been a report of this kind of separation using the one‐test method. According to the results of the forced degradation, formoterol fumarate dihydrate was sensitive to acid–base conditions, and budesonide was sensitive to base and photolysis. Based on the development and validation results, this method is stability‐indicating. It is a unique test method, and the economic cost of the technique is significantly less and user‐friendly.

## Conflicts of Interest

The authors declare no conflicts of interest.

## Data Availability

Data sharing does not apply to this article as no new data were created.
